# The impact of shorter prehospital transport times on outcomes in patients with abdominal vascular injuries

**DOI:** 10.1186/1752-2897-7-11

**Published:** 2013-12-21

**Authors:** Chad G Ball, Brian H Williams, Clarisse Tallah, Jeffrey P Salomone, David V Feliciano

**Affiliations:** 1 Departments of Surgery, University of Calgary, Foothills Medical Center, Calgary, Alberta, Canada; 2 Departments of Surgery, University of Texas – Southwestern Medical School, Parkland Memorial Hospital, Dallas, TX, USA; 3 Departments of Surgery, Emory University, Grady Memorial Hospital, Atlanta, GA, USA; 4 Departments of Surgery, Indiana University, Indianapolis, IN, USA; 5 Foothills Medical Centre, 1403 – 29 Street NW, Calgary, Alberta, Canada

**Keywords:** Trauma, Abdominal vascular, Injuries, Prehospital, Transport, Time

## Abstract

**Background:**

Most deaths in patients with abdominal vascular injuries (ABVI) are caused by exsanguination and irreversible shock. Therefore, time to definitive hemorrhage control is an important factor affecting survival. The study goals were: (1) document current outcomes in patients with ABVI, and (2) compare outcomes to those from the era preceding improvements in an urban prehospital system.

**Methods:**

A retrospective review of all patients with ABVI at an urban level 1 trauma center was completed. Patients injured prior to prehospital transport improvements (1991–1994) were compared to those following a reduction in transport times (1995–2004).

**Results:**

Of 388 patients, 70 (18%) arrived prior to prehospital improvements (1991–1994). Patient/injury demographics were similar in both groups (age, sex, penetrating mechanism; p > 0.05). The number of patients presenting with ABVI increased (23 vs. 35 per year; p < 0.05) concurrent to a reduction in transport times (27 vs. 20 minutes; p < 0.05). Patients were more frequently unstable (63% vs. 91%; p < 0.05). Regardless of the specific vessel, mortality increased (37% vs. 67%; p < 0.05) following prehospital improvements.

**Conclusions:**

A reduction in urban transport times resulted in an increase in (1) the number of patients arriving with abdominal vascular injuries, (2) the proportion presenting in physiologic extremis, and (3) overall mortality.

## Introduction

Abdominal vascular injuries refer to the disruption of major midline, mesenteric, retroperitoneal, and/or portal blood vessels.^1^ Although the incidence of abdominal vascular trauma varies depending on mechanism of injury (5% blunt to 25% penetrating) [1-4], they remain among the most lethal of all injuries [1-6]. Rapid operative exposure, damage control, definitive repair, and the sequential treatment of concurrent injuries are challenging for even the most experienced surgeons [7-9].

Because most deaths related to abdominal vascular injuries are a direct result of exsanguination and irreversible shock [1-10], the time interval between injury and operative control of ongoing hemorrhage remains a dominant variable in patient survival [11-13]. Although multiple factors contribute to this interval, prehospital transport and delays within the emergency department (ED) represent two potentially modifiable variables. For example, patients transported by non-emergency medical services (EMS) have been shown to arrive at the hospital earlier after their injuries [14]. Furthermore, prolonged stays in the ED clearly delay control of hemorrhage and result in an increased mortality. In essence, any factor related to ongoing blood loss and shock will profoundly affect survival [1-18].

This project was initiated after the anecdotal observation that despite clinical consistency amongst highly trained, double-boarded trauma surgeons employed at a high volume center, the mortality rate associated with abdominal vascular injuries appeared to increase with time. This occurred concurrently to an alteration in the regional urban prehospital transport system. The primary aims were therefore to (1) document current outcomes in patients with abdominal vascular injuries, and (2) compare these outcomes to the years preceding improvements in an urban EMS transport system.

## Material and methods

A retrospective cohort study comparing all adults (≥ 16 years) who had abdominal vascular injuries at a high volume, level one urban trauma center in Atlanta, Georgia, were compared in two eras. Era 1 (1991–1994) was defined by multiple prehospital providers. Era 2 (1995–2004) was characterized by improvements in the EMS system that led to consolidation of 3 out of 4 (exception was DeKalb county) ambulance zones under a single provider (Grady Memorial Hospital [GMH] EMS system). Previous to this change, multiple and variable EMS providers had been transporting patients to GMH from each of these zones. By consolidating this system, ensuring both quality control measures and algorithmic care, as well as a dominant emphasis on “scoop and run” of all penetrating injured patients, overall care could be ensured with minimal variability. These principles were consistent throughout era 2. This resulted in complete oversight and direction by the Emory University Departments of Surgery and Emergency Medicine at Grady Memorial Hospital. Members of the departments of Surgery and Emergency Medicine, Atlanta Police Service, GMH EMS, coroner, and nursing leads also met on a monthly basis in a multidisciplinary format to review all traumatic deaths, as well as discuss any additional pre-hospital, police or other issues of concern. Quality control remained paramount at all times and was particularly dominant at maintaining as short an interval as possible between injury and arrival at GMH. One of the techniques that helps ensure this delivery was strategic placement of EMS providers (i.e. ambulances) safely near urban areas that were known to generate significant volumes of penetrating injuries at late times of the day and night. This ‘holding pattern’ reduces overall transport times by minimizing the time from 911 call to the arrival of EMS providers. This retrospective project was approved by our Institutional Ethics Review Board in 2008.

Clinical methodology for the treatment of patients with abdominal vascular injuries was consistent between eras. Principles included: (1) early operative intervention for hemorrhage control (goal of ≤10 minutes in the trauma bay); (2) early vascular control followed by ligation, temporary intravascular shunt, or repair); and (3) traditional blood banking (i.e. preceded the implementation of damage control resuscitation) [17,19,20]. Patients who lost their vital signs within the trauma bay were included in the analysis. Patients who died prior to arrival at the trauma center were excluded. Hypotension and hemodynamic instability were defined as sBP < 90 mmHg.

The year 2004 was utilized as the study interval stop date because shortly thereafter, alterations in resuscitation strategy became much more common at GMH (the use of less crystalloid, hypotensive resuscitation, and more blood products that were eventually packaged as the Massive Transfusion Protocol).

Data sources included patient paper charts, Department of Surgery morbidity and mortality database, and computer-based laboratory information. Comparative analyses were conducted using Stata version 10.0 (Stata Corp., College Station, TX). Normally distributed variables were reported as means compared using students’ *t*-test. Differences in proportions for categorical data required Fisher’s exact or Chi-square tests. A two-sided p-value of ≤ 0.05 represented statistical significance.

## Results

Over the 13-year study period, 388 patients presented to the Emory University Trauma Service at Grady Memorial Hospital with abdominal vascular injuries. Both groups (Eras 1 and 2) were similar with regard to patient and injury characteristics (Table 1). This included relatively similar distributions of associated injuries (p > 0.05). With the onset of era 2, both scene (19 vs. 12 minutes; p = 0.01) and transport (27 vs. 20 minutes; p = 0.01) times decreased significantly. This was associated with an increase in the total number of patients with abdominal vascular injuries received by the trauma center (23 vs. 35 per year)(p = 0.02), as well as an increase in the rate of hemodymanic instability upon presentation (63% vs. 91%; p < 0.001).

**Table 1:** Patient and injury characteristic comparison between two eras

	**Era 1 (1991–1994)**	**Era 2 (1995–2004)**
Volume	70 (23/year)	318 (35/year)*
Age (mean)	31	32
Sex (male)	93%	94%
Mechanism (penetrating)	95%	94%
Gunshot wounds	91%	94%
Scene time (mean)	19 minutes	12 minutes*
Base deficit (mean)	-9	-11
Hypotensive at presentation	63%	91%*
EMS transport time (mean)	27 minutes	20 minutes*
Associated injuries per patient	1.7	1.9
Time in ED (mean)	25.4 minutes	14.1 minutes*
RBC transfused in O.R. (mean)	13.4 units	14.8 units
Mortality	37%	67%*

Legend:

* = p < 0.05.

Hypotensive = <90 mmHg sBP.

ED = emergency department.

RBC = red blood cells.

O.R. = operating room.

Era 2 had a significantly higher mortality rate (37% vs. 67%) amongst all patients with abdominal vascular injuries (p < 0.0001). This significant increase was consistent across all subtypes of abdominal vascular injuries: aorta (36% vs. 93%), iliac vessels (36% vs. 56%), visceral arteries (29% vs. 61%), inferior vena cava (48% vs. 67%), renal vein (33% vs. 75%), portal vein (40% vs. 73%), and splenic vein (40% vs. 78%).

Although comparison data were not available for era 1, patients in era 2 had a mean Injury Severity Score of 26 and mean age of 26 years. The number of patients who died within the trauma bay (i.e. at arrival) was also consistent between eras (4 and 5 per year in Eras 1 and 2 respectively). All patients who did not die in the trauma bay underwent an operative intervention with no difference across eras (100% laparotomy and 21% thoracotomy rates). Of the thoracotomies, 12% were performed within the ED.

## Discussion

It has been evident for decades that to improve morbidity and mortality in patients with major abdominal vascular injuries, the time from injury to hemorrhage control must be shortened [1-18]. In theory, rapid prehospital transport offers the hospital-based surgeon an earlier opportunity to arrest ongoing hemorrhage, replace lost blood volume and coagulation factors, and correct hypothermia. Given that patients need to be transferred from the scene of injury to definitive care at a trauma center, prehospital transport time is an essential *quality indicator* for the overall care of these patients. Whether transported by non-EMS family and friends [14], or highly trained paramedics [15], rapid transfer to a trauma center with surgeons skilled at stopping massive hemorrhage is a critical factor in survival of the injured patient [1-18].

The recent widespread implementation of damage control resuscitation, and its associated rapid administration of blood products to prevent and/or address early coagulopathies, has significantly altered overall survival in most major trauma centers [19-21]. Consequently, massive transfusion protocols have become a significant area of injury-related research [19-21]. Because similar transfusion strategies were employed across both eras in this study however, the data are not confounded by this recent change in management. More specifically, this data set precedes the introduction of a massive transfusion protocol at our center [20]. As a result, the observed increase in overall mortality (associated with shorter prehospital times) is likely specific to the issue of transport times. This finding is particularly relevant given that multiple publications have stated a need to focus research on injury prevention and prehospital care after observing no significant improvement in survival over decades of care amongst patients with abdominal vascular trauma [11,22].

With a consolidation and reorganization of an urban prehospital EMS system, the total volume of patients with abdominal vascular injuries received at Grady Memorial Hospital increased significantly (34% increase) in era 2. Given that one of the dominant aims of the EMS system was to improve efficiency, it is not surprising that the scene and overall transport times to the trauma center were reduced dramatically as well (37% and 26% decreases respectively). As a result, Grady received significantly more patients in physiologic extremis (63% to 91%). Given the consistent clinical practice and internal critique by the same group of trauma surgeons at this institution, as well as the lack of variability in patient or injury characteristics across eras, the link between faster scene/prehospital transport and subsequent increased mortality in patients with abdominal vascular injuries, appears direct. This is further supported by the actual decrease in time spent within the ED itself in the second era, and therefore the surgeons’ commitment to rapid transport to the operating theater. It should also be noted that a component of non-modifiable scene time related to security and safety of the EMS crews surrounding scenes of gunshot violence. Prolonged scene times were typically because the police or tactical teams had not yet secured the scene.

Although the link between prehospital transport times and subsequent mortality in patients with abdominal vascular injuries is strong and likely causal, this study has multiple inherent limitations. First, it is retrospective and therefore the possibility of bias cannot be eliminated. Second, despite a close working and administrative relationship between GMH EMS and the trauma surgeons, EMS transport times were still reliant on self-reporting. Third, despite the implied conclusion that transport times may have been ‘too fast’ and therefore delivered pre-morbid patients exhibiting physiologic exhaustion who can not be salvaged despite experienced surgical care, the authors could not detect a specific threshold or tipping point where this conclusion was statistically or practically significant. Finally, this study was also limited by a lack of reliable access to data from the 2 county coroners over the entire study interval to effectively evaluate prehospital traumatic deaths.

## Conclusions

In summary, shorter prehospital times in patients who would have previously died at the scene, or en route to the hospital, appear to result in increased mortality among patients with major abdominal vascular injuries. Although prehospital transport times, and trauma center associated 30-day mortality endpoints are vitally important quality indices, regional variables must also be taken into account. In addition, future studies on such patients will account for the influence of “damage control resuscitation”.

## Competing interests

CGB, BHW, CT, JPS and DVF declare no competing interests.

## Authors’ contributions

Study conception, data acquisition, data analysis, manuscript writing and final review was completed by CGB, BHW, JPS and DVF. Data acquisition was also completed by CT. All authors read and approved the final manuscript.
